# The role of human milk fats in shaping neonatal development and the early life gut microbiota

**DOI:** 10.20517/mrr.2023.09

**Published:** 2023-03-31

**Authors:** David Seki, Theresa Errerd, Lindsay J. Hall

**Affiliations:** ^1^Chair of Intestinal Microbiome, School of Life Sciences, ZIEL-Institute for Food & Health, Technical University of Munich, Freising 85354, Germany.; ^2^Gut Microbes & Health, Quadram Institute Bioscience, Norwich Research Park, Norwich NR4 7UQ, UK.; ^3^Norwich Medical School, University of East Anglia, Norwich Research Park, Norwich NR4 7TJ, UK.

**Keywords:** Early life, gut microbiota, breast milk, lipids, fat, metabolism

## Abstract

Human breast milk (HBM) is the main source of nutrition for neonates across the critical early-life developmental period. The highest demand for energy is due to rapid neurophysiological expansion post-delivery, which is largely met by human milk lipids (HMLs). These HMLs also play a prebiotic role and potentially promote the growth of certain commensal bacteria, which, via HML digestion, supports the additional transfer of energy to the infant. In tandem, HMLs can also exert bactericidal effects against a variety of opportunistic pathogens, which contributes to overall colonisation resistance. Such interactions are pivotal for sustaining homeostatic relationships between microorganisms and their hosts. However, the underlying molecular mechanisms governing these interactions remain poorly understood. This review will explore the current research landscape with respect to HMLs, including compositional considerations and impact on the early life gut microbiota. Recent papers in this field will also be discussed, including a final perspective on current knowledge gaps and potential next research steps for these important but understudied breast milk components.

## INTRODUCTION

Human breast milk (HBM) is pivotal for nutrition, immunological priming, neurodevelopmental support, and gut microbiome establishment in newborn infants. The mechanisms by which HBM impacts these key host responses are varied and include hormones for the regulation of digestion^[1]^, infant appetite^[2]^, and multiple pro- and anti-inflammatory cytokines to support and train the largely naive infant immune system^[3]^. HBM is considered the gold standard in infant nutrition, which is highly variable between lactating individuals^[4]^ and generally seems temporally and conditionally suited to match the respective infants’ needs^[5]^. One of the most studied nutritional substrates are human milk oligosaccharides (HMOs), which pass undigested into the infant colon where they can act as prebiotic (i.e., selective) nutrient sources for certain microbial genera such as Bifidobacterium. Fermentation of these HMOs then provides key metabolites to the growing infant and the wider microbiota^[6]^. Another key nutritional component of HBM are human milk lipids (HMLs), which are a major source of energy for infants, with an average content of ~40 g/L breast milk during the initial semester post-delivery^[7]^. HMLs also include several compounds that are essential for infant development. For example, phosphorylated lipids, glycosylated lipids, short- and long-chain fatty acids, polyunsaturated fatty acids^[8]^, and several fat-soluble vitamins^[9]^, which are all important for cognitive and immune system development^[10]^, bone growth^[11]^, anti-oxidation^[12]^, and establishment of gastrointestinal tract (GIT) mucus^[13]^. It is becoming increasingly clear that the GIT microbiota also participates in the degradation of HMLs, which like HMOs, may influence the initial assembly of specific microbial members and communities. Thus, HMLs may indirectly affect infant health via interactions between the host, bacteria, and their shared diet. However, in comparison to HMOs, our understanding concerning the microbial utilisation of HMLs is limited.

### The milk fat globule: core and membrane

Milk fat globules (MFGs) are functionally versatile droplets that are secreted into human milk via secretory cells of the mammary gland alveoli during lactation^[14]^. Their main nutritional purpose is to passage a triglyceride-rich core through the infants’ digestive tract. Although there is already a significant microbial ecosystem in the initial days post-birth^[15]^, and many colonisers are reportedly capable of incorporating dietary lipids^[16]^, the nutrient-rich core remains available for the infant as the milk fat globule membranes (MFGMs) protect the fat droplet from microbial digestion. MFGMs are heterogeneous and change throughout lactation, both regarding overall quantity and composition^[17]^. In general, MFGs are coated by two such membranes: a monolayer coating the triglyceride-rich core, and an overlying bi-layer membrane. Both membranes include proteins, phospholipids, sphingolipids, gangliosides, choline, sialic acid, and cholesterol, whereby the outer layer contains most of the glycolipids found in human milk (HM)^[8]^. Most of these glycolipids can be classified as gangliosides with a ceramide lipid chain anchoring respective combinations of oligosaccharides and sialic acids to the MFGM^[18]^. Notably, gangliosides have been shown to be highly important for both cerebral^[19]^ and enteral^[20]^ development, growth, communication, and differentiation of colonocytes^[21]^, and gut-associated immune cells^[22]^. The most abundant gangliosides in human milk are monosialoganglioside-3 (GM-3) and disialoganglioside-3 (GD-3)^[23]^. GD-3 is very abundant in colostrum, while GM-3 increases in abundance at later time points^[24]^. Gangliosides, in particular, have been shown in many cases to protect against gastrointestinal pathogens via inhibition of various toxins^[25]^, and GM-3 and GD-3 can act as decoy receptors for rotavirus^[26]^ and influenza viruses^[27]^, respectively. Indirectly, gangliosides exert anti-inflammatory effects on the developing immune system via influencing dendritic cell maturation^[28]^ and downstream stimulation of the hosts’ own intestinal IgA production^[22]^. However, gangliosides are not antagonistic to all gastrointestinal microorganisms. Previous work has indicated that the dominant and keystone early-life bacterial gut microbiota member *Bifidobacterium* can grow in the presence of GM-3 and GD-3 while simultaneously lowering ganglioside levels *in vitro*, which is suggestive of possible incorporation or conversion. Notably, different bifidobacterial species have different preferences. *Bifidobacterium bifidum* is very efficient in removing GD-3 by employing extracellular mechanisms, while *Bifidobacterium longum* subsp*. infantis* degrades GM-3 intracellularly, with overall lower glycosidase activity^[29]^. These observations indicate that gangliosides support the establishment of commensal microbiota during early-life.

The MFGM is equipped with many other compounds that, such as gangliosides, are interpretable as bioactive, as summarised in Table 1. Phosphatidylethanolamines (PEs) are integral to the outer MFG bilayer membrane, and while they are important for human cell proliferation during early life, some bacteria of the *Clostridium* and *Enterococcus* genera were shown to use ethanolamine as a source of carbon and nitrogen^[30]^. Furthermore, phosphatidylserine has implications for brain development in infants^[31]^ and was shown to induce major shifts in Bacillota (Firmicutes): Bacteroidota (Bacteroidetes) ratio in human gut microbiomes^[32]^. For strictly anaerobic representatives of the *Clostridia* and *Veillonella* genera, it was shown that they can use phosphatidylserine as a substrate to catalyse phosphatidylethanolamine and plasmenylethanolamine, both by employing respective phospholipid decarboxylase activities^[33]^, a function that is commonly found among many lactic acid bacteria found in the human gut^[34]^. Furthermore, phosphatidylcholine is a major membrane-forming phospholipid in eukaryotes, with an estimated presence of 15% in the bacterial kingdom^[35]^. As a provided substrate, it can exert a prebiotic function on *B. longum* subsp. *infantis*, which, by translating the compound to 1,2-sn-diacylglycerols, affects the regulation of colonic mucus production. However, conflicting evidence from *in vitro* studies indicates that *Clostridia* and Enterobacteriaceae could also be involved in phosphatidylcholine metabolism, which decreases the abundance of *Bifidobacterium* spp. due to competition for the substrate^[36]^. The outer MFG membrane also contains most of the sphingomyelins, for which many bactericidal activities against human opportunistic pathogens are described^[37]^, thereby greatly increasing colonisation resistance. Sphingomyelins were observed to increase gut barrier function, thereby decreasing the chances of translocation of intestinal bacteria during inflammation^[38]^. Dietary sphingomyelins were also observed to significantly reduce inflammatory cytokine levels in the circulation of mice^[39]^. However, sphingomyelins have also been observed to act as binding sites for toxins of the enteropathogen *Helicobacter pylori*^[40]^. However, sphingosine, a hydrolytic product of sphingomyelin, reportedly showed general bactericidal activity in the GIT^[41]^.

**Table 1:** Summary of bioactive MFGM components

**Lipid**	**Description**	**Role for Microbiome**	**Citation**
Phosphatidylethanolamine	Inner membrane lipid, important for cell proliferation and differentiation by regulation of immunological pathways. Degraded by phosphodiesterases to yield glycerol and ethanolamine	Certain intestinal bacteria including several pathogenic species such as *Clostridium*, *Enterococcus*, *Escherichia* and *Salmonella* catabolise ethanolamine as a major carbon and/or N source with the aid of ethanolamine utilisation proteins	[30,110,111]
Phosphatidylserine	Inner membrane lipid, responsible for the induction of apoptosis, carrier of Docosahexaenoic acid	Phosphatidylserine was observed to decrease the ratio of Bacillota (Firmicutes) to Bacteroidota (Bacteroidetes)	[32,112,113]
Phosphatidylinositol	Inner membrane lipid, important for cell signalling and activation of immunological pathways. Cell signalling, activation of Akt (1)	Phosphatidylinositol was shown to exert active bursting action on the protoplasts of *Bacillus megaterium*	[114,115]
Cholesterol	Found in inner and outer membrane, responsible for the structural maintenance of membranes, compartmentalization of membrane proteins, and serves as a substrate for bile acids, vitamin D, hormones and oxysterols	Several studies on germ-free animal models showed evidence of microbial involvement in cholesterol and bile metabolism	[8,116-118]
Phosphatidylcholine	Outer membrane lipid, important for membrane structure, lipoprotein assembly, and secretion	*Bifidobacterium longum subsp. infantis* was observed to utilize phosphatidylcholine to produce 1,2-sn-Diacylglycerols (DAG), which are involved in the regulation of colonic mucosal proliferation	[36,112]
Sphingomyelin	Metabolized to ceramide and sphingosine. Important for vascular development and immunological modifications	General bactericidal activities	[37,119-121]
Cerebrosides	Cerebrosides are major glycosphingolipids of human milk. These are glycolipids with a Galactose/Glucose moiety	Protect the procedure of digestion and gut-mucus integrity	[122,123]
Gangliosides	Gangliosides are glycosphingolipids consisting of a hydrophobic ceramide and a hydrophilic oligosaccharide chain. Seminal involvement for cognitive development and immunological modulation	Often described as putative decoys that enhance colonisation resistance against opportunistic pathogens	[19-22]
Monoacylglycerols	Hydrolysis of dietary triacylglycerols by endogenous lipases produces sn-2 monoacylglycerols	General bactericidal activities	[37,124]
Saturated fatty acids	Some dietary fatty acids are converted to biologically active metabolites by enzymes not only by the host but also by gastrointestinal bacteria	Bacteria can incorporate extracellular fatty acids into membrane lipids	[125,126]
Triacylglycerols	Diverse set of lipids found in the core of the MFG. Mainly consists of stearic, palmitic, oleic, linoleic, myristic and lauric acid	Some metabolic products of the acids (Monoglycerides) can have an inactivating effect on bacteria	[8,127]
Stearic acid	The high concentration of stearic acid in brain grey matter suggests that this fatty acid has an important role in neural function	General modulatory effects on gut microbiota	[43,128,129]
Palmitic acid	Used for energy metabolism and the synthesis of bioactive lipids	Higher proportions of palmitic acid in infant formula were observed to increase faecal *Lactobacillus* and *Bifidobacterium* levels	[45,124]
Oleic acid	Used for energy storage and metabolism, can alter cell membrane fluidity	Was observed as beneficial for growth of several *Lactobacillus* species	[46,130]
Linoleic acid	Involved in functions for skin barrier maintenance, a precursor to Arachidonic acid, and competes with n-3 fatty acid metabolism. Described as one of the most abundant and active fatty acids in protection from infections	Microbial conversion of linoleic acid into conjugated linoleic acids reportedly contributes to gut health	[131-134]
Myristic acid	Myristic acid is directly involved in post-translational protein changes and mechanisms that control important metabolic processes in the human body	Abundance of myristic acid was associated with *Bacteroides*, Enterobacteriaceae, *Veillonella, Streptococcus*, and *Clostridium* abundances in infant gut microbiota	[85,135]
Lauric acid	One of the most active fatty acids in protection from infections, makes up 5% of milk fatty acids	Lauric acid has significant antimicrobial activity against Gram-positive bacteria	[133,136]

The core of MFGs consists of a pool of triacylglycerols (TAGs) that is composed of several saturated fatty acids, including stearic, palmitic, oleic, linoleic, myristic, and lauric acids^[8]^. These by themselves interact with colonising microbiota, but especially during their initial hydrolysis, monoacylglycerols are generated, constituting prominent bactericidal bi-products ^[42]^. Stearic acid was found in high concentrations in infant brain grey matter, suggesting important implications for neurogenesis^[43]^. Furthermore, stearic acid, similar to palmitic acid, forms crystallite surfaces that display bactericidal activity against *Pseudomonas aeruginosa* and *Staphylococcus aureus*^[44]^, both relevant pathogens during human early life. Greater proportions of palmitic acid in the pool of triacylglycerides, for instance, were associated with higher levels of fecal *Lactobacillus* and *Bifidobacterium* in neonates^[45]^. Similarly, oleic acid was observed as beneficial for *Lactobacillus* spp.^[46]^, while lauric acid was shown to exert antimicrobial activity against *Cutibacterium acnes*^[47]^. Lastly, freely-available myristic acid is involved in the post-translational folding of proteins in humans^[48]^ and can inhibit the activity of some bacterial ATP-binding cassette (ABC) transporters, for instance, observed for bacillus multidrug-resistance ATP (BmrA) of *Bacillus subtilis (B. subtilis)*. The bmrA gene encodes an ABC half-transporter which, besides many different substrates, also transports cervimycin out of the cell, thereby rendering *B. subtilis* resistant to the antibiotic. In this case, freely-available myristic acid was observed to have an inhibitory effect on the respective ATPase and BmrA transport activity, thereby rendering the *B. subtilis* unviable^[49]^. Whether this is the case for other bacterial ABC transporters, such as LmrA or MsbA, remains to be elucidated. Furthermore, the accessibility of TAGs for hydrolysis is highly dependent on the structural integrity of the MFG during digestion^[50]^. Cholesterols are mainly responsible for the structural maintenance of both membranes but also serve as the substrate for bile acids, vitamin D, hormones, and oxysterols^[8]^. Recently, it was shown that *Clostridia* can metabolise cholesterol to coprostanol^[51]^, which escapes hepatic recirculation as it is not reabsorbed by colonocytes. Since cholesterol forms the backbone of bile-acid production, this has massive consequences for downstream absorption of lipids emulsions and implied interactions with gut microbiota during stabilisation and digestion of TAG-cores.

### Interactions of gut bacteria with the lipid emulsion during digestion

Human breast milk is considered the optimal food for the growth and development of healthy infants, as well as pivotal for the initial establishment of human gut microbiota. However, many infants are not exclusively fed breast milk during the initial months post-delivery, and previous work has indicated that formula-fed infants are more susceptible to diarrhea, pneumonia, and sepsis, which may be due to a lack of immunological support and colonisation resistance against pathogenic microorganisms provided by breast milk^[52]^. Indeed, receipt of breast milk was shown to be the most significant factor associated with infant gut microbiome composition, leading to an increased abundance and prolonged occurrence of bacteria of the genus *Bifidobacterium*, while cessation of breast milk was observed to be associated with a premature establishment of Bacillota instead^[53]^. However, it remains poorly understood how HMLs interact with intestinal microbiota during early-life succession. It is believed that the developing central and peripheral nervous systems account for the largest fraction of energy demand and expenditure during infancy^[54]^. Approximately half of this energy demand is met by digestible HMLs, such as TAGs^[55]^. The MFGM passes a TAG-rich core through the digestive tract in the presence of gastrointestinal bacteria. For neurons to receive this energy, homeostasis between the immune system and the microbiome is favourable^[56]^ during the digestion of a TAG emulsion. Questions remain as to where in the gut TAGs and other lipids are preferentially absorbed and under which circumstances colonising bacteria aid in absorption or become opportunistic scavengers of released nutrients.

Digestion of MFGs starts in the stomach, where gastric proteases begin to hydrolyse MFGM-bound proteins at low pH^[57]^. This partially destabilises the membrane and releases nutritious fat while simultaneously releasing sphingomyelins and cholesterol stabilise the coagulate, enabling adherence of lipases secreted from gastric mucus^[58]^. Gastric absorption of lipids is of higher relevance shortly post-delivery, as duodenal absorption is deficient due to the initial lack of bile acids and pancreatic lipases in the duodenum and onwards^[59]^. Bile acids are synthesised from cholesterol in the liver^[60]^, and once their synthesis is steadily established, primary bile acids are secreted from the gallbladder in dependence on ingested cholesterol: phosphatidylcholine ratios^[61]^. The primary bile acid profile in infants predominantly consists of cholic acid (CA) and chenodeoxycholic acid (CDCA), with a greater proportion of CA and its conjugates than CDCA and its conjugates^[62]^. These (conjugated) primary bile acids are key for the degradation of MFGs in the small intestine, where they ensure the removal of lipolytic products from the oil-water interface as surface-active molecules, coordinate micellar solubilisation, and stabilise lipid droplets against aggregation^[63]^. They furthermore stimulate the activity of lipases such as the bile salt-stimulated lipase (BSSL)^[64]^. Interestingly, BSSL is also produced in mammary glands and seeded via breast-feeding, while other lipases are only produced in the pancreas, such as pancreatic lipase-related proteins (PLRP) and pancreatic triglyceride lipase (PTL)^[57]^. Around 95% of all bile acids reabsorbed in the distal ileum enter hepatic recirculation^[65]^. The remaining, however, are subjected to microbial translation into secondary bile acids via microbial deconjugation, oxidation, epimerisation, 7-dehydroxylation, esterification, and desulfation. To do so, GIT microbes employ bile salt hydrolases (BSHs) in the presence of taurine or glycine to deconjugate primary bile acids^[66]^, a process that largely takes place in the small intestine and results in the hydrolysis of amide bonds in primary bile acids and leads to the release of free amino acids^[67]^. Generally, the microbiome from duodenum to ileum is phylogenetically less diverse and has lower biomass in total compared to the colon^[68]^. Bacterial genera commonly found in the infant small intestine include Lactobacillus, *Clostridium*, *Staphylococcus*, *Streptococcus*, *Bacteroides*, and *Bifidobacterium*^[69]^, many of which reportedly show respective BSH activity^[70]^. Currently, only a few three-dimensional structures of the BSH enzyme have been reported, including those of bacteria that are prevalent initial colonisers of the infant gut, such as *Bifidobacterium longum*^[71]^, *Enterococcus faecalis*^[72]^, and *Clostridium perfringens*^[73]^. While each is similar in topology, they display different catalytic efficiencies and substrate preferences^[74]^. It is well known that some of these bacteria establish as commensals in the human gut, while others may be detrimental to health when overly abundant, with a “disturbed” microbial composition potentially being funnelled via key actions of strain-specific BSH activities. Importantly, secondary bile acids escape hepatic recirculation, which reportedly, in turn, decreases cholesterol absorption and enhances its fecal excretion via modulation of farnesoid X receptor (FXR) signalling^[75]^. Microbial modulation of bile acid profiles has been linked to inflammatory bowel disease (IBD), with related FXR modifications as an underlying mechanism of gut barrier destabilisation^[76]^. Furthermore, microbial bile acid deconjugation was shown to involve immunological modifications, whereby ω-muricholic acid (ω-MCA) and 3β-hydroxydeoxycholic acid (isoDCA) in particular have been shown to stimulate dendritic cell recruitment and increase the frequency of Foxp3^+^ T regulatory cells^[77]^. However, it remains unclear which species are responsible for the given transformations.

### Temporal & incidental variability of human milk lipid composition

The size of the MFG and its lipid composition varies across the lactation period and is reflective of the needs of the infant^[78]^. Generally, a slight increase in the size of MFGs as well as total milk fat content was observed with the time of lactation^[79]^, likely to meet the increased caloric needs. However, MFG size has been shown to be surprisingly large in colostrum during the first two days post-delivery^[80]^, presumably as an adaption to the immature digestive tract and enteric immune system of the newborn. Phosphatidylcholine, phosphatidylethanolamine, and sphingomyelin contribute up to 40% of all MFGM phospholipids, which are subject to intra-individual variation, especially during early lactation, with concentrations ranging from 140 mg/L to 410 mg/L^[81]^, reflective of the plasticity of the MFGM during early life. *Lactobacillus* species have been shown to either incorporate or coat themselves with milk-derived phospholipids in a species-dependent manner, whereby they increase their surface electronegativity, which results in increased adherence to epithelial cells^[82]^. Species of the genus *Lactobacillus* are well-known commensals of the small intestine with many anti-inflammatory properties, and their successful establishment in the GIT is believed to contribute to the colonisation resistance against enteric pathogens^[83]^. Furthermore, phospholipids, as reviewed in detail elsewhere^[84]^, are important metabolites for intestinal cell integrity and maturation, and disruptions in sphingolipid metabolism were previously implied in the pathogenesis of preterm necrotising enterocolitis^[85]^. Interestingly, cholesterol concentrations appear to decrease during lactation as well^[86]^, which may have implications for bile acid production. Alongside changes in lipid composition, researchers repeatedly find reoccurring patterns of microbial succession during early life. In general, these include a transition of dominance from facultative anaerobic bacteria to a fully anaerobic lifestyle in the late phases of colonisation^[87]^. Under anaerobic conditions, intestinal bacteria ferment dietary carbohydrates and produce short-chain fatty acid (SCFA) end-products such as acetate, butyrate, and lactate, the composition of which varies depending on underlying microbial fingerprints^[88]^. SCFAs have many important interactions with the human host, including the importance of differentiation of dendritic cells^[89]^, as well as the promotion of mucus secretion and epithelial barrier integrity^[90]^. Interestingly, SCFAs are found in HM^[91]^, presumably to compensate for the initial lack of intestinal SCFA production in infant microbiota.

### Infant sex and socioeconomic status are involved in HM composition and early life microbiome establishment

Collective evidence suggests that the composite of HBM is personalised in order to secure optimal developmental conditions for the infant^[92]^. This highlights the necessity of future research to study mother-infant pairs in order to gain a better understanding of infant nutrition, including the role of HMLs, and early-life gut microbiome establishment. However, non-stochastic sources of variability, such as the difference of sex, provide partial explanations for the observed variance, as metabolic requirements between male and female newborns diverge^[93]^, implying disparities for BM absorption^[94]^ and microbiome establishment^[95]^, which should not be overlooked in future research planning. It was recently highlighted that human milk provides sex-specific growth advantages, even implying the existence of sex-specific micronutrients^[96]^. Indeed, there are several observations of sexual dimorphism and its obvious connection to nutrition. Overall, male and female growth rates differ^[97]^, and given that the majority of energy is delivered via HMLs, it is implied that HML composition and absorption may differ according to infant sex, while indeed, mothers of male infants produce BM with a higher energy content that mothers of female infants^[98]^. However, little is known whether microbiome-affecting lipids of the MFGM differ in dependency on infant sex, while several studies indicate there are potential sex-dependent differences in gut microbiota at different stages post-delivery. For example, it was reported that male premature infants have less rich microbiota with higher numbers of *Enterobacteriales*, as compared to female premature infants who show higher numbers of *Clostridiales*^[99]^, while another study reported on elevated abundances of *Bacteroides* spp. in female infants^[100]^. Also, there is strong evidence indicating that male infants are at higher risk for morbidities when challenged by perinatal complications^[101]^, but the underlying causes are not well-researched and practical guidelines for differential nutritional strategies are lacking. Maternal diet has furthermore been linked to BML contents and related growth of offspring^[102]^. Socioeconomic status furthermore is linked to the human diet^[103]^ and, therefore, partially underlies the HM content of lactating mothers. While the relationship is complex, obesity as well as malnourishment manifest in association with poverty^[104]^. It was shown that overall milk lipid contents are negatively associated with the BMI of Congolese mothers^[105]^, and HM fatty acid composition, especially levels of long-chain polyunsaturated fatty acids, were related to the socioeconomic status of Iraqi mothers^[106]^. Furthermore, it is well known that infant sex and socioeconomic status interactively define milk fat concentrations, given that mothers of sufficient socioeconomic status produce milk richer in fat for male offspring, while mothers of lower socioeconomic status produce milk richer in fat for female offspring^[107]^. However, it is not well understood how this affects the content of MFGMs and related downstream effects on microbiome establishment.

### Conclusion and future prospects of HML research

A balanced establishment of early-life gut microbiota is seminal for health throughout life. Diet heavily influences this succession, and the MFGM represents a largely overlooked interface for cross-communication between establishing microbiota and the developing infant. The MFGM contains a selective repertoire of molecules to strengthen colonisation success for human commensal bacteria and colonisation resistance against opportunistic pathogens, while simultaneously delivering a major fraction of energy supply through the digestive tract. In order to understand the various effects of HMLs on early life microbiome establishment, constituent parts of the MFGM and their effects on particular microorganisms during digestion of human milk is a key area for future research. For example, stable-isotope probing (SIP) techniques are extensively discussed for general application in the inquiry of human microbiomes^[108]^ and could be employed for the detection of utilisation of respective MFGM components by particular microorganisms of the establishing infant gut microbiome. Thereby, mechanisms underlying pathogenicity or colonisation resistance could be identified, described, and attributed to respective microorganisms. Diarrhea remains a major cause of child mortality^[109]^, and neurophysiological impairments following premature birth have been linked to aberrant development of the enteric microbiota^[88]^. Therefore, globally many infants and their families would benefit from such additional research to improve colonisation resistance against pathogenic microorganisms and to improve outcomes against serious infections with novel therapeutic options while concurrently reducing antibiotic usage, which is also linked to the antimicrobial resistance crisis. Furthermore, our understanding of early-life microbiota is biased, as most sequencing efforts have focused on samples from high-income countries. Therefore, future microbiota profiling and dietary mechanistic studies should be broadened to capture a more global and true perspective on infant gut microbial communities and the diversity and impact of HMLs.

## DECLARATIONS

### Copyright

© The Author (s) 2023.
